# Monthly pork price forecasting method based on Census X12-GM(1,1) combination model

**DOI:** 10.1371/journal.pone.0251436

**Published:** 2021-05-11

**Authors:** Chuansheng Wang, Zhihua Sun

**Affiliations:** 1 Capital University of Economics and Business, Beijing, China; 2 School of Management Engineering, Capital University of Economics and Business, Beijing, China; South China University of Technology, CHINA

## Abstract

**Background:**

In recent years, the price of pork in China continues to fluctuate at a high level. The forecast of pork price becomes more important. Single prediction models are often used for this work, but they are not accurate enough. This paper proposes a new method based on Census X12-GM(1,1) combination model.

**Methods:**

Monthly pork price data from January 2014 to December 2020 were obtained from the State Statistics Bureau(Mainland China). Census X12 model was adopted to get the long-term trend factor, business cycle change factor and seasonal factor of pork price data before September 2020. GM (1,1) model was used to fit and predict the long-term trend factor and business cycle change factor. The fitting and forecasting values of GM(1,1) were multiplied by the seasonal factor and empirical seasonal factor individually to obtain the fitting and forecasting values of the original monthly pork price series.

**Results:**

The expression of GM(1,1) model for fitting and forecasting long-term trend factor and and business cycle change factor was *X*^(1)^(*k*) = −1704.80*e*^−0.022(*k*−1)^ + 1742.36. Empirical seasonal factor of predicted values was 1.002 Using Census X12-GM(1,1) method, the final forecast values of pork price from July 2020 to December 2020 were 34.75, 33.98, 33.23, 32.50, 31.78 and 31.08 respectively. Compared with ARIMA, GM(1,1) and Holt-Winters models, Root mean square error (RMSE), mean absolute percentage error (MAPE) and mean absolute error (MAE) of Census X12-GM(1,1) method was the lowest on forecasting part.

**Conclusions:**

Compared with other single model, Census X12-GM(1,1) method has better prediction accuracy for monthly pork price series. The monthly pork price predicted by Census X12-GM(1,1) method can be used as an important reference for stakeholders.

## Introduction

In many country, pork is one of the daily necessities for most ordinary families. According to statistics, China needs about 54 million tons of pork every year, but the data released by National Bureau of statistics(Mainland China), pork output in 2019 is only 42.55 million tons, which shows that there is still a big gap between this quantity and the actual demand. Although pig breeding enterprises and individuals have also applied some innovative technologies to expand the production of pigs, the imbalance between supply and demand of pork is still an important factor affecting the price of pork in China [1]. The rising price of pork in 2019 is also an important factor leading to the continuous rise of China CPI [2]. The stability and controllability of pork price is not only related to the living standard of ordinary people, but also reflects the level of national governance in a sense.

Through the analysis of pork price trend, government departments can formulate strategies according to local conditions [3], such as encouraging breeding, putting government reserve pork into the market, or taking restrictive measures. Understanding the change of pork price in advance can also make farmers, middlemen and final consumers prepare for the follow-up plan [4]. For example, farmers can decide whether to expand the scale, middlemen can decide whether to increase or reduce the quantity of pork stocks, and final consumers may also consider whether to replace pork with other food. It takes about six months for a pig to become a marketable pork commodity from birth, that is to say, the price changes in the next six months are most meaningful for stakeholders [5].

Nowadays, there are many mathematical models that can be used to fit and predict time series data [6]. The monthly pork price data are a kind of typical time series. The models commonly used to fit and forecast time series include ARIMA model, grey system model, Holt winters exponential smoothing model and so on. With the development of artificial intelligence technology, various kinds of neural network technology are also used to forecast time series [7–9]. In addition, in order to analyze the factors that affect the change of time series, Census X12 model is used to decompose the seasonal and long-term change trend of time series [10–12]. In order to improve the accuracy of time series prediction, multiple model combination method is also widely used [13–16].

GM (1,1) model is one of the most important models for time series prediction [17]. It is one of the core contents of the grey system theory established by Professor Deng Julong in 1982 [18]. It describes the dynamic changes of time series by establishing a first-order linear ordinary differential equation. Its characteristic is that it can use a small amount of data to model and predict the series data. GM (1,1) model can be used to predict a wide range of time series, such as traffic data prediction [19–22], financial data prediction [23, 24], agricultural data prediction, weather data, geological disaster data, disease prevention and control data, etc [25–28]. At the same time, in order to further improve the prediction accuracy, GM (1,1) model is also combined with other models [29–32].

In this paper, a new model combination method based on Census X12 model and GM (1,1) model was used to fit and forecast monthly pork price series. By comparing the prediction results of this method with other models, it was proved that X12-GM(1,1) model combination method has higher prediction accuracy. In the process of forecasting monthly pork price time series using Census X12-GM(1,1) method, a calculation parameter called empirical seasonal factor was constructed. When Census X12-GM(1,1) method is applied to other types of time series, the calculation formula of the empirical seasonal factor may be different.

## Materials and methods

### Materials source

The original monthly pork price data from January 2014 to December 2020 were collected from the National Bureau of statistics(Mainland China). We divided the sample data into two parts, the data from January 2014 to June 2020 were used for model fitting, and the data from July 2020 to December 2020 were used to evaluate the accuracy of model prediction.

### Census X12 model

The traditional time series analysis method divides the fluctuation of time series into four factors: long-term trend change factor (T), seasonal change factor (S), business cycle change factor (C) and irregular change factor (I). Census X12 model is a decomposition method of seasonal factor of time series. Based on the different degree of independence between the factors, the model can be divided into additive decomposition model and multiplicative decomposition model.

The basic principle of Census X12 is to decompose the long-term trend change factor and business cycle change factor by the centralized moving weighted average method. The seasonal change factor is obtained by dividing the monthly data average of the time series by the total average, and the irregular change factor is obtained by dividing the original series by the long-term trend change factor, business cycle change factor and seasonal change factor. The algorithm of Census X12 multiplication model is shown as follows:

**Step one**: Initial estimate of seasonal adjustment

After centralization of 12 items moving average,

$$\begin{array}{c} TC_{t}^{\left(1\right)}=(\frac{1}{2}Y_{t-6}+Y_{t-5}+\cdots +Y_{t}+\cdots +Y_{t+5}+\frac{1}{2}Y_{t+6})/12 \end{array}$$

$$\begin{array}{c} {\left(SI\right)}_{t}^{\left(1\right)}=\frac{Y_{t}}{TC_{t}^{\left(1\right)}} \end{array}$$

By 3 × 3 moving average process, the initial estimation of seasonal factor *S*_*t*_ has

$$\begin{array}{c} {\hat{S}}_{t}^{\left(1\right)}=[{\left(SI\right)}_{t-24}^{\left(1\right)}+2{\left(SI\right)}_{t-12}^{\left(1\right)}+3{\left(SI\right)}_{t}^{\left(1\right)}+2{\left(SI\right)}_{t+12}^{\left(1\right)}+{\left(SI\right)}_{t+24}^{\left(1\right)}]/9 \end{array}$$

After eliminating the residual trend of season factor,

$$\begin{array}{c} S_{t}^{\left(1\right)}={\hat{S}}_{t}^{\left(1\right)}-({\hat{S}}_{t-6}^{\left(1\right)}+2{\hat{S}}_{t-5}^{\left(1\right)}+\cdots +2{\hat{S}}_{t+5}^{\left(1\right)}+{\hat{S}}_{t+6}^{\left(1\right)})/24 \end{array}$$

So, after seasonal adjustment, the initial estimation has

$$\begin{array}{c} {\left(TC\cdot I\right)}_{t}^{\left(1\right)}=\frac{Y_{t}}{S_{t}^{\left(1\right)}} \end{array}$$

**Step two**: Calculation of tentative trend cycle factor and final season factor

Using “Henderson” moving average formula, tentative trend cycle factor can be calculated. That is

$$\begin{array}{c} TC_{t}^{\left(2\right)}=\sum_{j=-H}^{H}h_{j}^{\left(2H+1\right)}{\left(TC\cdot I\right)}_{t+j}^{\left(1\right)} \end{array}$$

Among them, $h_{j}^{\left(2H+1\right)}$ is “Henderson” moving average coefficient. Then

$$\begin{array}{c} {\left(SI\right)}_{t}^{\left(2\right)}=\frac{Y_{t}}{TC_{t}^{\left(2\right)}} \end{array}$$

By 3 × 5 moving average process, tentative season factor can be calculated. That is

$$\begin{array}{c} \begin{array}{c} {\hat{S}}_{t}^{\left(2\right)}=[{\left(SI\right)}_{t-36}^{\left(2\right)}+2{\left(SI\right)}_{t-24}^{\left(2\right)}+3{\left(SI\right)}_{t-12}^{\left(2\right)}+3{\left(SI\right)}_{t}^{\left(2\right)}\\ +3{\left(SI\right)}_{t+12}^{\left(2\right)}+2{\left(SI\right)}_{t+24}^{\left(2\right)}+{\left(SI\right)}_{t+36}^{\left(2\right)}]/15 \end{array} \end{array}$$

So, final season factor can be drawn. That is

$$\begin{array}{c} S_{t}^{\left(2\right)}={\hat{S}}_{t}^{\left(2\right)}-({\hat{S}}_{t-6}^{\left(2\right)}+2{\hat{S}}_{t-5}^{\left(2\right)}+\cdots +2{\hat{S}}_{t+5}^{\left(2\right)}+{\hat{S}}_{t+6}^{\left(2\right)})/24 \end{array}$$

At this point, second estimate results of seasonal adjustment shows as follows

$$\begin{array}{c} {\left(TC\cdot I\right)}_{t}^{\left(2\right)}=\frac{Y_{t}}{S_{t}^{\left(2\right)}} \end{array}$$

**Step three**: Calculation of final trend cycle factor and season factor

Using “Henderson” moving average fourmula, final trend cycle factor can be calculated. That is

$$\begin{array}{c} TC_{t}^{\left(3\right)}=\sum_{j=-H}^{H}h_{j}^{\left(2H+1\right)}{\left(TC\cdot I\right)}_{t+j}^{\left(2\right)} \end{array}$$

So final season factor has

$$\begin{array}{c} I_{t}^{\left(3\right)}=\frac{{\left(TC\cdot I\right)}_{t}^{\left(2\right)}}{TC_{t}^{\left(3\right)}} \end{array}$$

So far, the final decomposition multiplication model of series *Y*_*t*_ can be expressed as

$$\begin{array}{c} Y_{t}=TC_{t}^{\left(3\right)}\times S_{t}^{\left(2\right)}\times I_{t}^{\left(3\right)} \end{array}$$

### GM(1,1) model

The grey prediction theory was put forward by Chinese scholar Deng Julong in 1982. This method mainly aims at small samples with little data and missing information. In grey system theory, GM (1,1) model is the most basic time series prediction model. It can model and predict according to a small amount of information. The modeling process of GM (1,1) model is shown as follows:

**Step one**: The original sequence of GM (1,1) model was established.

That is

$$\begin{array}{c} X^{\left(0\right)}=\left(x^{\left(0\right)}\left(1\right),x^{\left(0\right)}\left(2\right),\ldots ,x^{\left(0\right)}\left(n\right)\right) \end{array}$$

**Step two**: Establishing one time accumulation generating sequence of the original sequence of GM (1,1) mode.

That is

$$\begin{array}{c} X^{\left(1\right)}=\left(x^{\left(1\right)}\left(1\right),x^{\left(1\right)}\left(2\right),\ldots ,x^{\left(1\right)}\left(n\right)\right) \end{array}$$

Among them,$x^{\left(1\right)}\left(k\right)=\sum_{i=1}^{k}x^{\left(0\right)}\left(i\right)$, k = 1,2,…,n, the purpose is to eliminate the contingency and instability of the original data.

**Step three**: The mean generation sequence of *X*^(1)^ is established as follows

$$\begin{array}{c} Z^{\left(1\right)}=\left(z^{\left(1\right)}\left(2\right),z^{\left(1\right)}\left(3\right),\ldots ,z^{\left(1\right)}\left(n\right)\right) \end{array}$$

Among them, *z*^(1)^(*k*) = 0.5 × (*z*^(1)^(*k*) + *z*^(1)^(*k* − 1), k = 1,2,…,n.

**Step four**: Constructing data matrix B and data vector Y, solving the values of development coefficient a and grey action quantity b.

*x*^(0)^(*k*) + *az*^(1)^(*k*) = *b* is the mean form of GM(1,1) model, and $\frac{dx^{\left(1\right)}}{dt}+ax^{\left(1\right)}=b$ is the whitening differential equation of GM(1,1) model.

$$\begin{array}{c} B=[\begin{array}{cc} -z^{\left(1\right)}\left(2\right) & 1\\ -z^{\left(1\right)}\left(3\right) & 1\\ \ldots & \ldots \\ -z^{\left(1\right)}\left(n\right) & 1 \end{array}],Y=[\begin{array}{c} x^{\left(0\right)}\left(2\right)\\ x^{\left(0\right)}\left(3\right)\\ \ldots \\ x^{\left(0\right)}\left(n\right) \end{array}] \end{array}$$

$$\begin{array}{c} {}[\begin{array}{c} a\\ b \end{array}]={\left(B^{T}B\right)}^{-1}B^{T}Y \end{array}$$

Therefore, the time response equation of the model is

$$\begin{array}{c} {\hat{x}}^{\left(1\right)}\left(k\right)=\left(x^{\left(0\right)}\left(1\right)-\frac{b}{a}\right)e^{-a(k-1)}+\frac{b}{a} \end{array}$$

If k = 1, then *x*^(1)^(1) = *x*^(0)^(1). So far, the original time series can be fitted and predicted by ${\hat{x}}^{\left(0\right)}\left(k\right)$. ${\hat{x}}^{\left(0\right)}\left(k\right)$ can be expressed as

$$\begin{array}{c} {\hat{x}}^{\left(0\right)}\left(k\right)={\hat{x}}^{\left(1\right)}\left(k\right)-{\hat{x}}^{\left(0\right)}\left(k-1\right),k=1,2,\ldots ,n \end{array}$$

Finally, Whether the GM (1,1) model can be applied to practical problems needs to be judged, and generally the average relative error, correlation degree, mean square error ratio and other indicators need to be verified.

### Census X12-GM(1,1) method

The algorithm of census X12-GM (1,1) method is divided into the following steps:

**Step one**: Acquisition of the original time series (*Y*_*t*_)

**Step two**: Factor decomposition of the original time series (*Y*_*t*_)

Census X12 model is used to decompose the component factors of the original time series (*Y*_*t*_). The result is that the original time series (*Y*_*t*_) is decomposed into long-term trend change factor, business cycle change factor (TC), seasonal change factor (S) and irregular change factor (I).

**Step three**: Fitting and forecasting of long-term change factor, business cycle cycle change factor (TC)

In this step, the GM(1,1) model is used. The fitting value and prediction value ($\hat{TC}$) of the long-term trend change factor and the business cycle change factor (TC) are obtained.

**Step four**: Multiplication operation of The fitting value and prediction value ($\hat{TC}$) and seasonal change factor (S)

It should be noted that the fitting value is multiplied by the seasonal factor of the original time series at the corresponding time point, and the predicted value is multiplied by a empirical value (*S*_*em*_) and the empirical value is calculated as follows:

$$\begin{array}{c} S_{em}=1+\frac{y_{n}-y_{n-1}}{10} \end{array}$$

*y*_*n*_ and *y*_*n*−1_ represent the last two values of the original sequence to be predicted.

**Finally**, the fitting value and prediction value (${\hat{Y}}_{t}$) of the original time series data (*Y*_*t*_) based on X12-GM(1,1) method are obtained.

**Fig 1:**
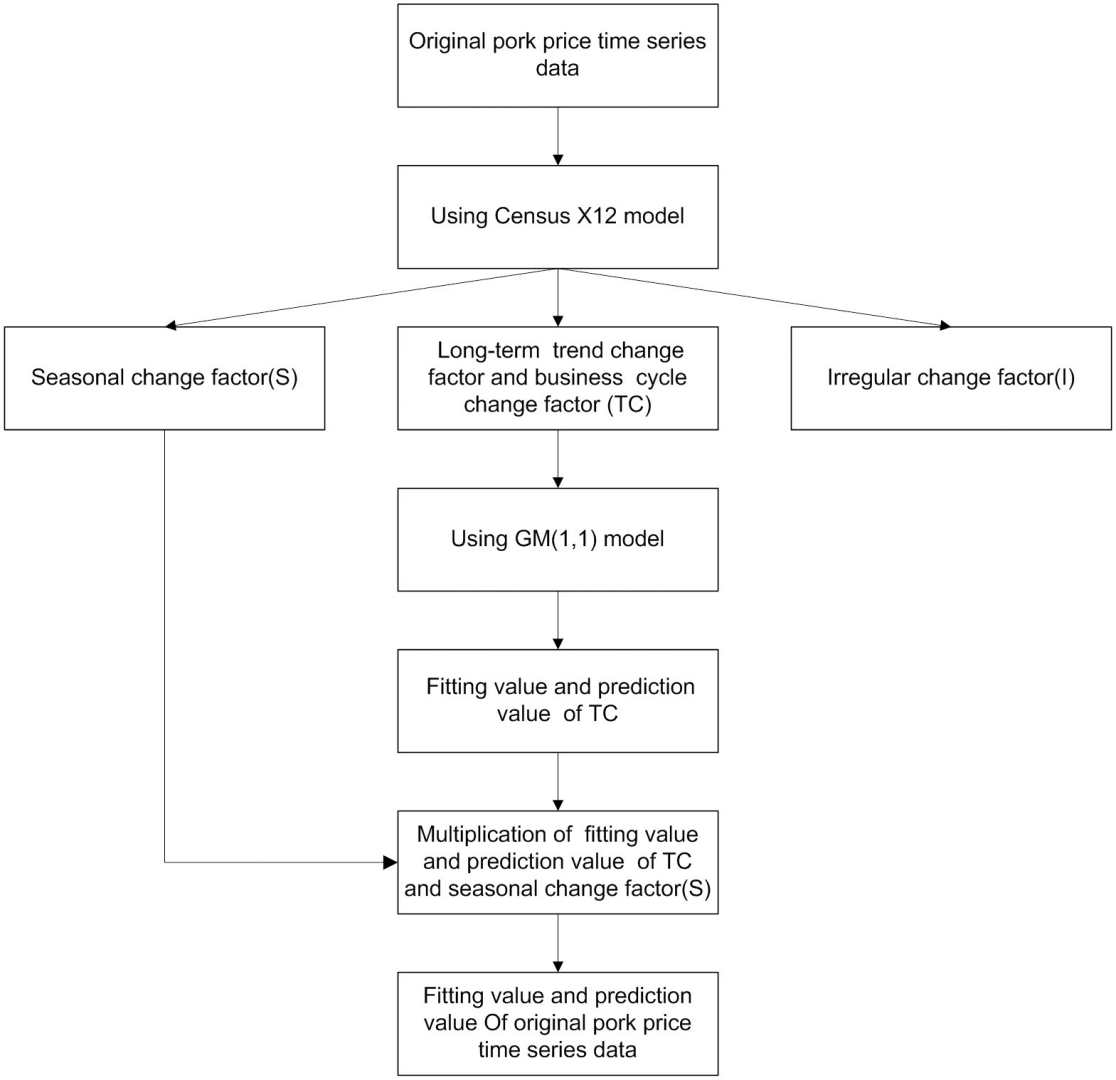
The algorithm flow of Census X12-GM(1,1) method.

The algorithm flow chart of Census X12-GM (1,1) method is shown in Fig 1.

### Comparison of prediction accuracy

In order to evaluate the prediction accuracy of different methods, this paper uses root mean square error (RMSE), mean absolute percentage error (MAPE), mean absolute error (MAE) as evaluation indexes [33, 34]. These indexes are espressed as:

$$\begin{array}{c} RMSE=\sqrt{\frac{1}{n}\sum_{i=1}^{n}{\left(y_{i}-\hat{y_{i}}\right)}^{2}} \end{array}$$

$$\begin{array}{c} MAPE=\sum_{i=1}^{n}\left|\frac{y_{i}-\hat{y_{i}}}{y_{i}}\right|\times \frac{100}{n} \end{array}$$

$$\begin{array}{c} MAE=\frac{1}{n}\sum_{i=1}^{n}\left|y_{i}-\hat{y_{i}}\right| \end{array}$$

### Data processing and analysis

Excel 2013 software was used to store the original monthly pork price time series from the National Bureau of statistics(Mainland China). Eview 9 software was used to build census X12 model and GSTA 7 software was used to build GM (1,1) model. The curve of fitting value and predicted value of Census X12-GM (1,1) method was also drawn by Eview 9 software.

## Results

### Overview of original monthly pork price time series

**Fig 2:**
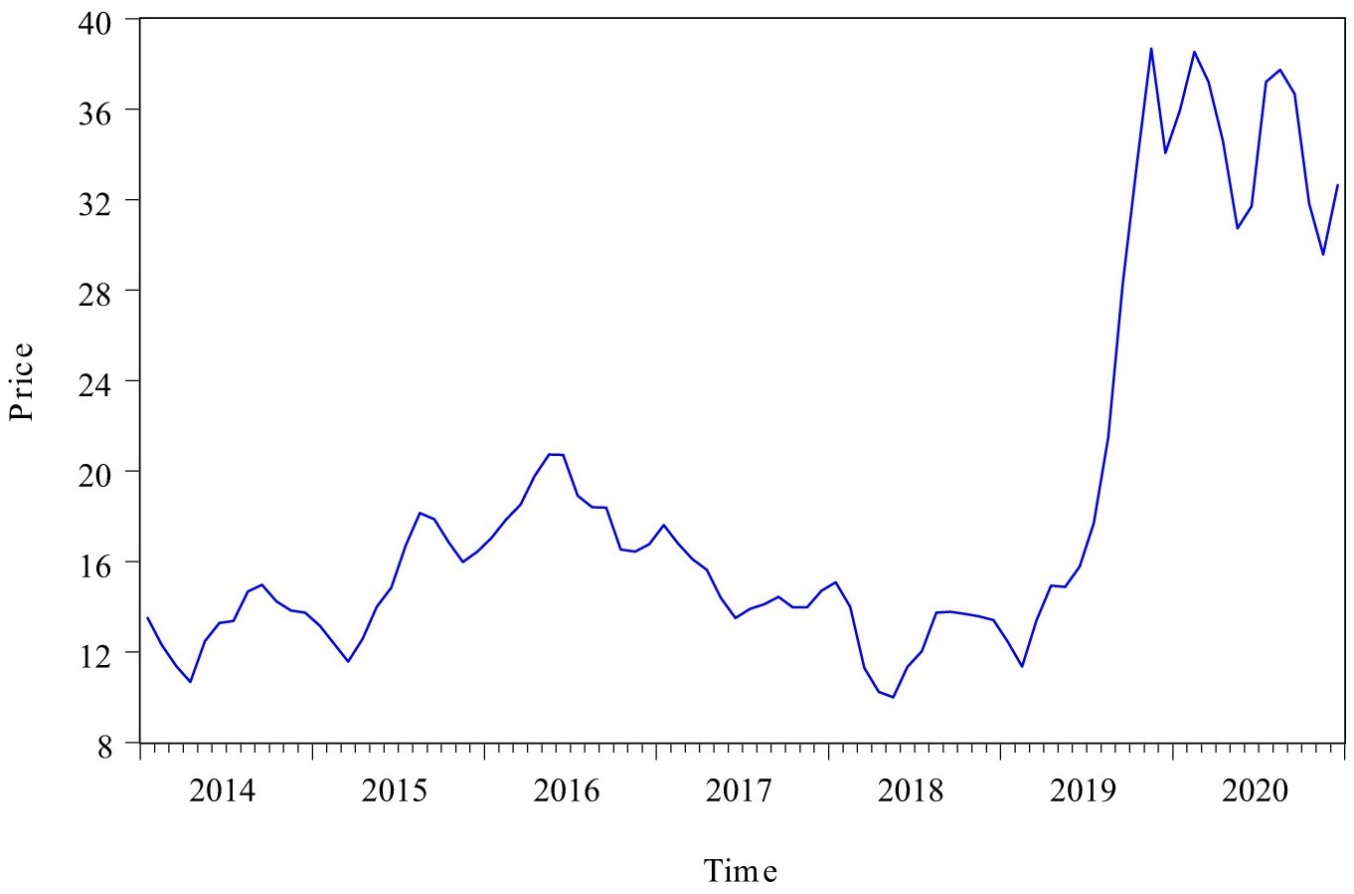
Monthly price of pork from January 2014 to December 2020.

A total of 84 numbers were collected to develop Census X12-GM(1,1) method. Fig 2 showed the change trend of the monthly pork price time series from the National Bureau of statistics(Mainland China). It could be seen from the figure that this time series had obvious business seasonal fluctuation and long-term trend characteristics. This time series had a relative peak from March 2015 to June 2017, and it started to rise from February 2019, and this trend was continued to November 2019. After November 2019, this time series began to fluctuate sharply at a high level.

### Acquisition of component factors using Census X12 model

**Fig 3:**
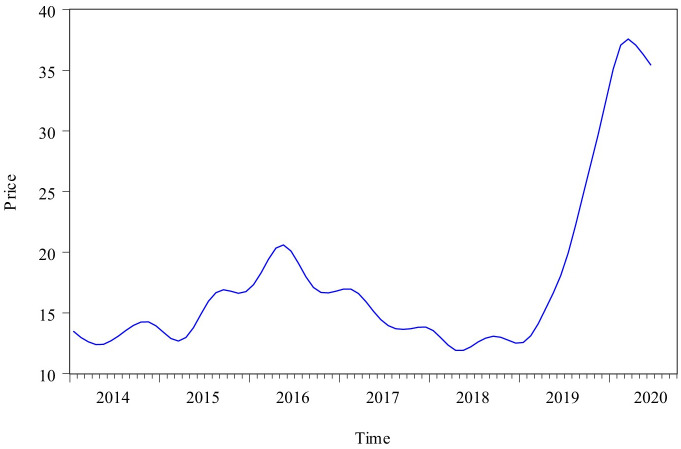
The curve of TC factor from January 2014 to June 2020.

**Fig 4:**
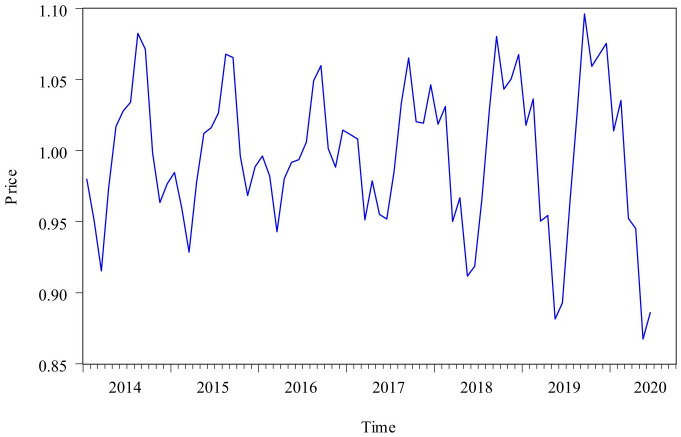
The curve of seasonal factor from January 2014 to June 2020.

**Fig 5:**
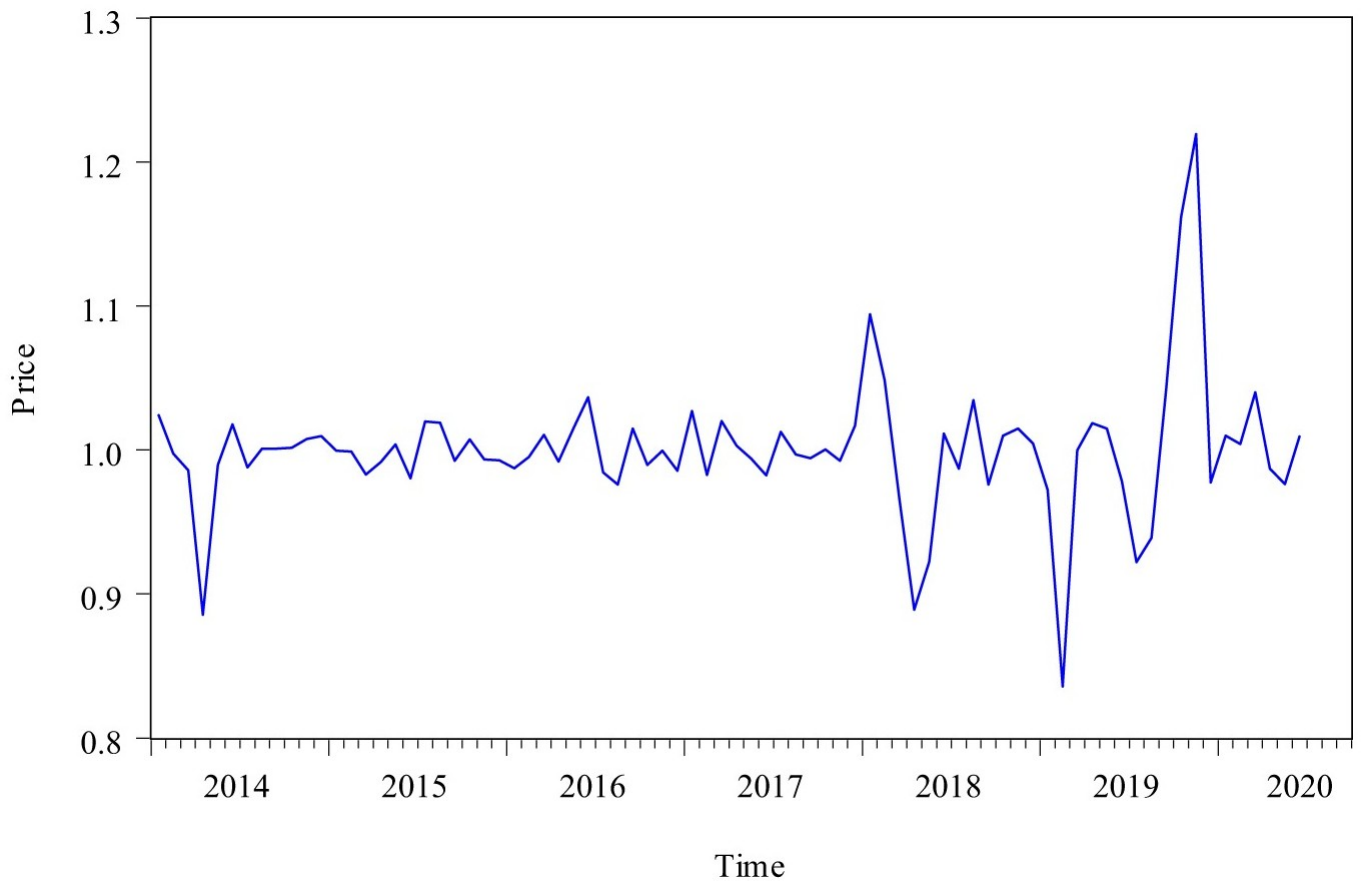
The curve of irregular factor from January 2014 to June 2020.

Census X12 model can be divided into multiplication model and addition model. This paper used Census X12 multiplication model to decompose the original monthly pork price time series from January 2014 to June 2020. The decomposing process did not consider the impact of traditional holidays on the data fluctuation. The decomposing results were as follows: Fig 3 showed the curve of long-term trend change factor and business cycle change factor (TC), Fig 4 showed the curve of seasonal factor, and Fig 5 showed the curve of irregular factor.

### Fitting and forecasting with GM (1,1) model

Next, the GM (1,1) model was built using the time series composed of long-term trend change factor and business cycle change factor (TC). Finally, we got the GM (1,1) model: *X*^(1)^(*k*) = −1704.80*e*^−0.022(*k*−1)^ + 1742.36. It should be noted that the model only used the long-term trend change factor and business cycle change factor (TC) of March 2020, April 2020, May 2020 and June 2020. There were two reasons for using these four data in modeling. On the one hand, the GM (1,1) model based on static data has a very good fitting effect, but the model fitting is not our key task. On the other hand, GM (1,1) model is more suitable for small sample and scarce data. Three to five sample points are enough [35]. The predicted values of long-term trend change factor and business cycle change factor (TC) obtained by GM (1,1) model were shown in Table 1.

**Table 1:** The predicted values of TC from July 2020 to December 2020.

Time	Jul	Aug	Sept	Oct	Nov	Dec
Predicted value	34.68	33.91	33.16	32.43	31.71	31.01

### Calculation of final fitting value and predicted value with Census X12-GM(1,1) method

Using Census X12 model, the seasonal factor of the original monthly pork price time series had be obtained. Using GM (1,1) model, the fitting value and predicted value of long-term trend change factor and business cycle change factor (TC) had be obtained. Census X12-GM (1,1) method was to multiply the above two kinds of data according to the corresponding time points. Here, It should be noted that the empirical seasonal factor(1.002) was multiplied by the predicted value. The empirical seasonal factor was obtained by special calculation. The final predicted value obtained by Census X12-GM(1,1) method was shown in Table 2.

**Table 2:** The final predicted values of the original series from July 2020 to December 2020.

Time	July	Aug	Sep	Oct	Nov	Dec
Predicted value	34.75	33.98	33.23	32.50	31.78	31.08

### Comparison of different methods

In order to compare the accuracy of different methods for forecasting the original monthly pork price time series, the original values of October 2020, November 2020 and December 2020 in the original monthly pork price time series and the predicted values of other methods include Census X12-GM(1,1) method, ARIMA model, GM (1,1) model and Holt-Winters model were shown in Table 3. Meanwhile, the prediction error indexes of the above four methods were also calculated and shown in Table 4. The above indexes proved that Census X12-GM (1,1) method had the best prediction performance for monthly pork price time series.

**Table 3:** Comparison of predicted value and actual value from July 2020 to December 2020.

Time	July	Aug	Sep	Oct	Nov	Dec
Actual value	37.20	37.73	36.67	31.83	29.57	32.63
Census X12-GM(1,1)	34.75	33.98	33.23	32.50	31.78	31.08
ARIMA	33.04	33.91	34.26	34.39	35.11	35.43
GM(1,1)	29.50	28.18	26.93	25.73	24.59	23.50
Holt-Winters	31.72	33.19	33.77	32.48	31.96	30.13

**Table 4:** Comparison of prediction error indexs.

Indexes	RMSE	MAPE	MAE
Census X12-GM(1,1)	2.5701	6.7058	2.3450
ARIMA	3.7146	10.5397	3.5483
GM(1,1)	8.0697	22.7596	7.8667
Holt-Winters	3.4505	8.7431	3.0767

### Prediction

Census X12-GM(1,1) method was used to obtain the time series data of pork price in January 2021, February 2021 and March 2021 (Table 5). The forecast data showed that the price of pork would decrease significantly in the next six months.

**Table 5:** The predicted values of Census X12-GM(1,1) method from January 2021 to June 2021.

Time	Jan	Feb	Mar	Apr	May	Jun
Predicted value	25.82	24.27	22.82	21.45	20.17	18.95

## Discussion

When the prediction results of multiple models was compared, some key information of other models was omitted. Next, the details of the other models will be shown.

When ARIMA model was used to fit the monthly pork price series, the normative modeling steps were carried out. Firstly, ADF (Augmented Dickey-Fuller test) was used to judge the stationarity of the original pork price series. After the first-order difference of the original series, the original pork price series met the requirements of stationarity. Next, ARIMA (0,1,1) and ARIMA (2,1,3) were created respectively according to the ACF (Autocorrelation Function) and PACF(Partial Autocorrelation Function) graph. Finally, by comprehensively judging the values of *R*^2^, AIC (Akaike Info Criterion) and SC (Schwarz Criterion), ARIMA (2,1,3) was chosen. Of course, the residual after fitting the original time series with ARIMA (2,1,3) model was also tested for correlation, the result showed that it was white noise.

The specific form of single GM (1,1) model used to directly fit the original monthly pork price time series was *X*^(1)^(*k*) = −768.93*e*^−0.045(*k*−1)^ + 806.13. The post test ratio (c) and small error probability (P) of the model are acceptable.

Holt-winters model is a kind of exponential smoothing method for fitting time series, which can be subdivided into seasonless model, additive model and multiplicative model. According to the actual situation of the data, we used its multiplicative model.its parameter values were automatically set as *α* = 1, *β* = 0.34, *γ* = 0 by the Eviews software.

In the process of using Census X12-GM(1,1) method to forecast monthly pork price, we used a parameter which was called an empirical seasonal factor. This parameter comed from the results of many experiments. For other practice series, the seasonal factors may be different, so it should be judged again according to the historical data. Census X12-GM(1,1) method only predicted the pork price data in the next six months. This is because the pig breeding cycle is about six months. It is also feasible to use Census X12-GM(1,1) method for longer time prediction, but the longer the time, the worse the accuracy.

## Conclusion

In this paper, several different methods were used to forecast monthly pork price data, including ARIMA model, GM (1,1) model, Holt winters model and Census X12-GM(1,1) method. By comparing RMSE, MAPE and MAE indexes, it was proved that Census X12-GM (1,1) method was more accurate. The prediction results of Census X12-GM(1,1) method can provide important reference for pig farmers, policy-making departments, middlemen and final consumers.

## Supporting information

S1 File The data of pork price from January 2014 to December 2020. (XLS)
